# Rethinking family-based obesity treatment

**DOI:** 10.1111/cob.12614

**Published:** 2023-08-02

**Authors:** Joseph A. Skelton, Mara Vitolins, Keeley J. Pratt, Leila Hamzi DeWitt, Sally G. Eagleton, Callie Brown

**Affiliations:** 1 Department of Pediatrics, Wake Forest University School of Medicine, Winston-Salem, North Carolina, USA; 2 Center for Prevention Science in Child and Family Health, Wake Forest University School of Medicine, Winston-Salem, North Carolina, USA; 3 Department of Epidemiology and Prevention, Wake Forest University School of Medicine, Winston-Salem, North Carolina, USA; 4 Department of Human Sciences, Human Development & Family Science Program, College of Education and Human Ecology, The Ohio State University, Columbus, Ohio, USA; 5 Department of Surgery, The Ohio State University Wexner Medical Center, Columbus, Ohio, USA; 6 Clinical and Translational Science Institute, Wake Forest University School of Medicine, Winston-Salem, North Carolina, USA

**Keywords:** family environment, family intervention, obesity, systems, weight management, youth

## Abstract

Emerging research in paediatric obesity has demonstrated that parental involvement in the weight management process can improve weight outcomes in children. Recent guidelines by the American Academy of Pediatrics note the importance of parent and family involvement in treatment. However, it is currently unknown if including the entire family in obesity treatment can supersede outcomes associated with participation of only one parent. Family Systems Theory (FST) provides the theoretical foundation for examining one’s healthy behaviours as they exist within the context of their family, defined by family dynamics. This narrative review aims to reconsider prior definitions of paediatric family-based management using the FST framework to be inclusive of family and household diversity and in doing so, inform research not only within weight management but also other domains of clinical care requiring family support or change. Applying FST to paediatric weight management highlights the link between family dynamics and paediatric obesity, demonstrating the association of dysfunctional family dynamics with more severe obesity. While family-based weight management remains the gold standard for treatment of paediatric obesity, more investigation is needed in expanding family-based interventions to impact entire families and potentially improve outcomes more broadly for overall family health and wellbeing.

## 1 | INTRODUCTION

Obesity prevalence remains unacceptably high, and during the COVID-19 pandemic, obesity appears to have worsened.^1^ Access to evidence-based treatment for obesity is increasingly recognized as critical in preventing unsafe, self-directed dieting behaviours.^2,3^ Recently, the American Academy of Pediatrics (AAP) released the ‘Clinical Practice Guidelines for the Evaluation and Treatment of Children and Adolescents with Obesity’ (CPG), updating and summarizing the evidence that have advanced to the point of being treatment guidelines (vs. expert recommendations).^4^ The CPG reviews the evidence behind behaviourally based treatment of obesity, and features newer medications, bariatric surgery and targets of behavioural change that are making headway in improving the health and weight status of individuals affected by excess weight, yet the majority of youth with obesity are not receiving evidence-based care, and effective outcomes from weight management requires a substantial time commitment from families and health care providers.^5^

In youth, it is recognized that the inclusion of family, particularly a parent, guardian or adult caregiver (for simplicity, referred to as parent herein), in the weight management process is of utmost importance.^6^ Starting in the 1980s, and continuing through today, inclusion of a parent showed improved weight outcomes for children.^7,8^ Intuitively this makes sense, as children depend on their parents for food, housing, transportation and overall support. In addition, parents are the primary role models of health behaviours for their children, demonstrating and establishing eating and activity habits. Further, parents establish rules pertaining to meals, sleep schedules and media use, and direct the utilisation of health care. In fact, research has termed ‘parents as exclusive agents of change’, in which the parent participating in weight management alone is equally efficacious in addressing child weight as the parent *and* the child participating together.^9,10^

While family-based treatment is considered the gold-standard for paediatric weight management, *whole* family participation is typically not being practiced. As any provider working with children will attest, typically only one parent attends clinical visits. This could be because of the diversity in family structures (multi-generational families, single parent households and blended families), dual-working parents or parents attempting to minimize time away from school and work. It is understandable and not surprising that all family members involved in caring for a child would find it difficult to attend the 26+ contact hours recommended by the United States Preventive Task Force to effectively treat childhood obesity.^4,5^ This raises the question, ‘Is treatment truly family-based if only one parent is participating, without siblings and other adults important to the health of the child?’ Many sentinel studies in this area clearly identify a participating parent (not parents, and typically women or mothers) and show positive outcomes,^7,8,11^ but it is not known how other household members are engaged in health behaviour change, or their awareness that the child and identified parent are participating.^12,13^ It is currently unknown if including the entire family in treatment can improve child outcomes over and above outcomes associated with the participation of one parent, and if it could improve adherence to treatment plans and sustainability of changes made. Further, to what degree can entire families participate in a treatment programme, and would outcomes be improved if they would?

The goal of family-based treatment is to change the health behaviours of the entire family and not focus solely on the child with obesity, as that could potentially harm the child by making them feel excluded from normal family routines and heighten challenges of self-esteem and bullying/teasing within families.^4^ When only parent–child dyads are included in treatment, clinicians are typically only observing the interactions of that dyad and relying on their self-reports of other household members’ behaviours. With questioning, more can be learned about the family and their health behaviours, particularly if others in the family have obesity or are attempting behaviour change. Many factors within a family could mediate the behaviour change process: what role does each family member play (who cooks, who shops for food, do they have similar parenting styles when it comes to discipline, schedule and rules); are there special circumstances that would impact families making changes, such as another family member with medical or behavioural issues (celiac disease, inflammatory bowel disease and autism spectrum disorder); what are work and school schedules; how is information from clinic/programme visits relayed to the rest of the family; what is the readiness to change of each family member; does the family have relationship or interpersonal challenges that could make changes in routines difficult; and how is treatment implemented differently within households based on family structure (i.e., one parent vs. two parents vs. blended families)? There are many aspects of the family and household that are important to consider when initiating paediatric weight management.

The overall purpose of this narrative review is to reconsider prior definitions of paediatric family-based weight management to be inclusive of the diversity of families and households that children reside. This type of inclusion should encompass additional household family members in treatment, consider the interpersonal dynamics among them that affect children’s outcomes, and finally, determine if treatment has potential ‘ripple effects’ or benefits to family members’ health status. Re-evaluating how families are assessed and included in clinical care settings may improve treatment approaches, identify new areas of intervention and personalize and individualize care for families. Exploration of the family system in clinical care may inform research in these areas to test ideas and assumptions. Further, it could impact clinical care outside of weight management, in any situation that requires changes family support or change.

## 2 | PAEDIATRIC OBESITY TREATMENT BECAME FAMILY-BASED

Medical writings on obesity in children from the 1940s can be easily found,^14^ and probably even earlier with greater effort and older technology (microfiche). In the 1970s, more publications discuss obesity in children,^15–17^ including one discussing using behavioural modification in treatment.^18^ With the increasing prevalence of obesity in the 1980s, more research and clinical approaches focussing on behavioural treatment of obesity can be seen from leaders in the field such as Wing,^19^ Epstein,^20,21^ Brownell,^7,22,23^ Israel^8,24^ and others. Much of this work began to define the role of parents and family in the treatment process. Israel et al.^24^ and Graves et al.^25^ studied parent roles in treatment, using parental problem-solving and training in parent management skills to successfully improve child weight. In addition to outlining the use of behaviour modification in treatment with Wadden,^23^ Brownell et al.^7^ demonstrated *how* including mothers in treatment with their children mattered, with mothers and children being treated separately (but in parallel) having greater success in weight loss than children alone and mothers and children together. The importance of parent involvement was solidified in Epstein and colleague’s classic studies that had 10 years of follow up, demonstrating superiority of parent inclusion in treatment.^26,27^ Flodmark et al.^28^ was also pursuing family-based treatment via family therapy (discussed later) during this same time, preventing the progression of severe obesity in children of 10–11 years of age. Lastly, Golan et al.^29^ and later Boutelle et al.^30,31^ studied having parents being the exclusive targets of intervention, with children not being involved in treatment. While this was primarily in younger children (<12 years of age), they demonstrated that parents were an important variable when approaching treatment in younger children. In an umbrella review of family-based weight management interventions, Chai et al.^32^ again surmised the effectiveness of including family members, and highlighted strategies of parenting, role modelling and encouragement of healthy diet and exercise in whole families.

As the rising prevalence of obesity began being referred to as an epidemic in the 1990s, there was increasing focus on the best approaches of treatment. The AAP developed and published expert committee recommendations in 1998,^33^ again in 2007,^34^ and newly released Clinical Practice Guidelines in 2023,^4^ which all referenced treatment being ‘family-based’. While this is unlikely to be the origin of the term ‘family-based’ in regards to paediatric weight management, it is highly referenced in standard of care. In these expert recommendations and guidelines, the studies of Brownell, Israel and Epstein are referenced, explaining ‘Clinicians should involve the family and all caregivers in the treatment programme’,^33^ including discussion of the importance of healthy parenting practices. The early work of the 1980s and 1990s demonstrating the inclusion of parents in the treatment process formed the basis of today’s recommendations of including the family in treatment, focusing on family-level habits and not just on the habits of the child.^4^

Revisiting the original studies (Brownell, Israel and Epstein) in which the term ‘family-based’ are often referenced, readers may question if ‘family-based’ is an inclusive description if only a parent was included as the representative of the family. This is not a fault or error of prior research; as it is quite common for a parent to grant permission for the child to participate and then consent to participate themselves. It may be that these studies are more dyad-based (parent–child) in nature versus being family-based interventions. While a focus on parenting, which was featured in some of the studies described, could impact a family, these were not whole-family interventions. Also, the focus within these studies, as is in clinical settings, was on family habits, not just the child’s habits, relying on the parent–child dyad to transfer these clinical activities to the home environment.

## 3 | RIPPLE-EFFECT OF TREATMENT

There is evidence that parents who participate in paediatric weight management programmes benefit as well, with improvements noted in their health behaviours and weight, in addition to improvement in their child’s weight.^11,31,35^ This is intuitive, as paediatric weight management trials and clinical programmes often utilize family-based approaches, and focus lifestyle changes on the entire family, not solely the child with obesity.

There is limited research with methodological challenges (i.e., short term and retrospective) which demonstrates that children with obesity whose parents are undergoing metabolic and bariatric surgery have improvements in weight, summarized in a recent review.^36^ There is far less, if any, evidence about changes in child weight and behaviour when parents are attempting non-surgical weight loss; a small feasibility study (*N* = 20) did show improvement in parent and child weight status with a commercial weight loss programme for adults at 8 weeks.^37^ A secondary analysis of parents participating in a weight management programme found their children with overweight and obesity overall had a decrease in their body mass index *z*-score, while children with a healthy weight had no change.^38^

While there are no readily available studies exploring the effect of weight management on siblings, there are a few studies showing associations of siblings’ weight and health behaviours in the general population but not in paediatric weight management programmes. Research led by Berge has noted weight and weight-related behaviour correlations between adolescents, their parents and siblings, finding greater correlation between siblings for some behaviours^39^; if the siblings were discordant on weight, parents employed potentially harmful, restrictive feeding practices on the child with excess weight.^40,41^ In a mixed-methods study, they also found differential weight talk within households, with siblings and fathers using more negative talk about weight, including teasing.^42^ These studies highlight the need to broaden the scope of interventions to include the rest of the family, both to possibly improve outcomes and prevent harm (weight talk).

Overall, there appears to be some evidence of a ‘ripple-effect’, with weight management (adult- and child-focused) improving the weight and health behaviours of others in the family, but it is far from definitive. While including a parent and child in treatment is likely to impact the rest of the family, there are many mediating factors. As an example, a study in a paediatric primary care setting found that only 58% of parents discussed weight management visits with other family members, and the likelihood of that was associated with family communication quality.^13^ Including only one parent attending weight management visits with a child is often more practical and feasible, but more effort could be made to facilitate information spread to other family members and to support the change process from clinic to home, as that ‘ripple effect’ is now dependent upon the family members attending clinic. An interesting review by Kitzmann and Beech^43^ assessed 31 family-based obesity studies, sorting them into categories based on having broad (parenting skills or family functioning) or narrow (nutrition and exercise) parent/family behaviour targets. Study designs and results were varied, leading the authors to ask how best to involve the parents in treatment, and if interventions should focus broadly on parenting skills and family dynamics, or more narrowly on health behaviours. Of particular note in the review, the 1993 study of Flodmark et al.^28^ is an example of successfully utilizing a family therapy approach, which Kitzmann and Beech classify as a broad focus on family functioning and parenting. Subsequent works by Flodmark, Nowicka and others have explored these deeper aspects of the family and obesity, with evidence of effectiveness.^44,45^

## 4 | FAMILY DYNAMICS AND OBESITY

There has been some discussion and investigation into family assessment and paediatric weight management, noting the limited use of the family studies field in this area.^46^ Pratt and Skelton^47^ discussed integrating Family Systems Theory (FST, discussed below) into family-based paediatric weight management to include the routine assessment of family dynamics along with health behaviours. Many aspects of family dynamics are pertinent to a child’s health behaviour changes, with family dynamics generally referring to relationships, interactions, roles and environment within a family. Often in paediatric weight management, dynamics are assessed through parenting styles, family functioning, family communication and family stress. Family dynamics (parent–child, sibling–sibling, parent–sibling, etc.) are important to consider, as dysfunctional relationships can lead to a negative household environment, making changing of routines and schedules more contentious. According to FST, family members should be flexible and adaptable to changing behaviours, rules and communication to adopt healthier behaviours to support the child in treatment. In clinical settings, whether working with a parent–child dyad or with multiple family members, relationships and communication are important considerations, and while clinicians may intuit some of these elements in a treatment visit, understanding their impact or influence on outcomes to date is unknown. There is some evidence that family dynamics and obesity in children are linked, with dysfunctional or impaired dynamics associated with more severe obesity,^48,49^ and better family functioning associated with better outcomes after bariatric surgery.^50^ In a brief review of the available literature, poor family functioning was associated with unhealthy weight-related behaviours and a higher child weight status.^51^ While nascent, existing evidence does highlight the importance of family dynamics, and the potential importance it has in paediatric weight management.

While the focus on family dynamics has been presented as a new approach to consider, others have been pursuing since the early 1990s. As detailed earlier, Flodmark et al.^28^ included family therapy in treatment. Nowicka and Flodmark^44^ detailed their use of family therapy in the treatment of childhood obesity, called Standard Obesity Family Therapy (SOFT), which focused more broadly on parenting, family functioning and overall family dynamics, noting the limited number of follow up sessions (three-to-four a year) and focus on family interactions. Their literature review in 2008 supports the use of family-focused treatment,^52^ including the use of behavioural interventions (cognitive behavioural therapy, etc.), and also highlighted the same Kitzmann and Beech review where the majority of obesity interventions focused on family health behaviours versus family function.^43^ While they have had a long-standing platform of focusing on family dynamics and obesity, there remains little mention of these treatment approaches in the new AAP CPG,^4^ reflecting the need to integrate this evidence into current interventions.

## 5 | FAMILY SYSTEMS THEORY

FST^53,54^ has been used to inform family assessment and treatment (most notably assessments based on the McMaster Model of Family Functioning^55,56^). FST is conceptualized and applied to clinical research and treatment in different formats (i.e., solution-focused therapy, cognitive and behavioural family therapy), but at its core, recognizes that individuals must be understood within the context of their family (Table 1).

The complexity of the family system is understandably difficult to apply to everyday clinical settings, but does have important ramifications for paediatric weight management in the context of family-based treatment. In particular, it can provide a framework for understanding the family represented by the likely scenario of a parent–child dyad attending treatment visits. Providers should ask questions regarding roles, relationships, communication, rules and structure within the home. Pratt and Skelton^47^ discussed an FST-informed approach to assess the families in paediatric weight management, which includes inquiring about household structure, rules, communication, behavioural control, responsiveness, involvement, strengths and barriers to change. Nowicka and Flodmark note their SOFT treatment is derived from FST.^44^ A systematic review of randomized trials only found two studies utilizing FST, with mixed results compared to traditional family-based therapy.^57^

## 6 | FAMILY-LEVEL INTERVENTIONS

Based on the review on family-level interventions in paediatric weight management by Kitzman and Beech^43^ and Sung-Chan et al.,^57^ it is not possible to determine if intervening with families beyond the participating parent–child dyad has an equal or increased positive effect to the child and other family members. However, other fields can offer some evidence about the benefits of extending treatment beyond the parent–child dyad, such as in the treatment of substance use disorders^58^ and type 1 diabetes,^59^ in which including individuals beyond the parent–child dyad led to improved outcomes.

Family meals, or meals eaten together at home with other family members, are an important family-level behaviour with inherent interpersonal interactions that should be considered in relation to children’s weight. Several studies, systematic reviews and meta-analyses have demonstrated positive associations between higher frequency of family meals and healthier children’s weight, dietary behaviours and practices,^60–62^ although the consistency and quality of the evidence varies.^63^ Further, the effects extend beyond nutritional habits, and positively influence other aspects of well-being, such as decreased substance use, violence, sexual activity,^64^ and mental and behavioural health issues^65^ and better family relationships.^66^ These findings speak towards a broader, simultaneously positive effect by identifying areas of family-level behaviours and dynamics linked to obesity, and intervening on those areas to potentially improve the health of the entire family, a different kind of ‘ripple effect’.

## 7 | REVISITING FAMILY-LEVEL INTERVENTIONS

Current family-based interventions provide evidence of positive impacts on child weight and health.^4,5^ Revisiting the early, formative literature, and the disparate studies accounting for family members in the household beyond the parent–child dyad, it could be worth exploring efforts to broaden interventions to account for and impact the broader family system. Briefly summarizing:

- There is some evidence of a ‘ripple effect’ in which children of parents pursuing weight management (particularly undergoing metabolic and bariatric surgery) and siblings of children in weight management improve their weight.
- Most evidence in paediatric weight management is based on *parent*–*child dyads* only.
- Parents of children who participate in family-based, paediatric weight management may improve their own weight (and potentially health), which often is precursory to positive changes in their child’s weight, indicating a *family intervention*, or a different form of the ‘ripple effect’; including additional family members may potentially magnify changes and impact.
- Evidence exists that *family dynamics* are associated with severe obesity in children.
- There is a theoretical basis for focusing on the *family system* and integrating behavioural and family systems theories into paediatric weight management.

There is a practical case to be made for extending interventions to focus on entire families, as more individuals can be impacted by a single intervention, versus separate interventions for children and adults. As seen in Golan and Boutelle’s parents as exclusive agents of change, intervening with a parent can improve the weight of a child.^30^ Conceptually combining that with the influence we see on parents’ weight when participating with their children, and the improvement in child weight and behaviours when adults undergo metabolic and bariatric surgery, there is the potential to improve the health of all family members in the household. Many evidence-based programmes, such as the Diabetes Prevention Program^67^ or Help Prevent Diabetes (HELP-PD),^68^ could be adapted to target the family system to improve family health habits, or adapting effective paediatric weight management programmes to better extend the ‘ripple effect’ to other members of the family.

It could be argued that existing paediatric weight management programmes, classified as family-based, already focus on family health habits, as evidenced by reports showing improvement in parent and child weight. But do these behaviourally based programmes, which use change techniques such as motivational interviewing, cognitive behavioural therapy, tracking and monitoring and goal setting, account for the complexities of the family system? Or for the complex nature of family dynamics: communication, parenting, family functioning, stress, rules, routines and schedules? While this may complicate a treatment approach, it also has the potential of spillover effects as seen in family meals, improving overall wellbeing and a decrease in other obesity-related and psychosocial risk factors. An approach that includes the entire household also has the potential for increasing family-centeredness of approaches, where standard health behaviours (consuming more fruits and vegetables, increasing exercise) may not implemented, but families are empowered with individualized, personalized ways to determine what can be done to improve their family dynamics in a way to optimize their health behaviours.

There are many practical challenges to this concept. More research is needed to determine best approaches, and in particular, which aspects of the family system have the potential to make the greatest impact on health and weight, which could differ between parent and child. This would require a move away from ‘weight-loss’ in a family, which can be quite different between adults and children, and a move towards health behaviour improvement, with weight change being a favourable side effect of more healthful behaviours in those with obesity (long-term goal). This, however, could prove frustrating for a parent or adolescent desiring weight loss (although much weight that is lost with short-term efforts are eventually regained). As with any health behaviour, and in particular weight management interventions, more focus is needed on safety, ensuring disordered eating and unhealthy weight control behaviours do not arise. However, recent expert guidelines assure that professional, behaviourally based weight management programmes protect against disordered eating in children.^4^ In clinical, non-research settings, defining the ‘patient’ for insurance purposes could be challenging; if focused on the family, is the parent the ‘patient’, or the child? Is this determined by who may have obesity or a comorbidity, and if both parent and child do, are they both considered the ‘patient?’ For cost-effective care, focusing insurance reimbursement on one individual is logical, although it presents challenges if the parent and child have different insurance providers, or one is commercial and one is government funded. This presents the opportunity to collaborate with primary care providers: a multidisciplinary programme can provide family-focused behavioural treatment, with medical management (comorbidity management and anti-obesity medications) being coordinated with the various primary care providers (paediatricians, family medicine and internists).

Other logistical challenges include finding ways to practically include additional family members in the household based on school and work schedules. Additionally, family members may have differing levels of interest in participating in a family-based treatment programme and readiness to make behavioural changes. More research is needed into inclusion via telehealth and mobile applications, group-based family sessions and opportunities for families to participate in after-hours or weekend programmes. There is also the possibility to focus on ‘parents as exclusive agents of change’ interventions, to better incorporate family-level interventions (i.e., improving family communications, relationships and schedules) to improve health behaviours of the family through the parents. Cultural adaptations of family-based treatments are an opportunity for innovation, but also require deep knowledge of families’ traditions and background, as well as greater diversity in the teams providing care. A systematic review in 2017 of prevention interventions noted the limited number of studies targeting diverse populations, including non-traditional families, although there were several focused on Hispanic and low-income families,^69^ while a more recent study found some disparities in family-based treatment by race and ethnicity.^70^ Finding ways to include extensions from paediatric weight management into families’ local communities may prove more advantageous, by engaging additional family members where they already are, such as local parks, social clubs, religious services and schools. Using community-engaged approaches in developing interventions will likely improve and facilitate cultural adaptations, address the gaps in the literature and advance health equity, as the recent AAP CPG notes the lack of evidence in this area.^4^

## 8 | CONCLUSIONS

Family-based weight management remain the gold standard for the treatment of paediatric obesity, in combination with advances in bariatric surgery and pharmacotherapy. With the new AAP CPG, there will be increasing interest and efforts to bring evidence-based treatment to children and families. There is the potential to extend these family-based interventions in a way to impact entire families, possibly improving outcomes for the child, siblings and parents, impacting families’ weight, health and wellbeing through more intentional focus on family dynamics and function. More research, experience and collaboration are needed to investigate these possibilities.

## Figures and Tables

**TABLE 1:** Brief summary of Family Systems Theory.^45,46^

Family is a complex system
Members of the family system are interconnected, and must be viewed as a whole
The behaviour of the system interacts with the environment as a feedback loop
The family determines who are members and sets boundaries
Families strive to maintain equilibrium, although they must evolve and change
Subsystems exist within the larger system, and can influence the larger family system
There are higher and lower levels within the family system, with higher orders causing change in lower orders

## References

[1] Jenssen BP, Kelly MK, Powell M, Bouchelle Z, Mayne SL, Fiks AG. COVID-19 and changes in child obesity. Pediatrics. 2021;147(5): e2021050123. doi:10.1542/peds.2021-050123 33653879

[2] Cardel MI, Newsome FA, Pearl RL, Patient-centered care for obesity: how health care providers can treat obesity while actively addressing weight stigma and eating disorder risk. J Acad Nutr Diet. 2022;122(6):1089–1098. 35033698 10.1016/j.jand.2022.01.004 PMC10056599

[3] Jebeile H, Gow ML, Baur LA, Garnett SP, Paxton SJ, Lister NB. Treatment of obesity, with a dietary component, and eating disorder risk in children and adolescents: a systematic review with meta-analysis. Obes Rev. 2019;20(9):1287–1298. 31131531 10.1111/obr.12866 PMC6851692

[4] Hampl SE, Hassink SG, Skinner AC, Clinical practice guideline for the evaluation and treatment of children and adolescents with obesity. Pediatrics. 2023;151(2):e2022060640. 36622115 10.1542/peds.2022-060640

[5] Whitlock EP, O’Connor EA, Williams SB, Beil TL, Lutz KW. Effectiveness of weight management interventions in children: a targeted systematic review for the USPSTF. Pediatrics. 2010;125(2):e396–e418. 20083531 10.1542/peds.2009-1955

[6] Spear BA, Barlow SE, Ervin C, Recommendations for treatment of child and adolescent overweight and obesity. Pediatrics. 2007;120-((suppl 4)):S254–S288. 18055654 10.1542/peds.2007-2329F

[7] Brownell KD, Kelman JH, Stunkard AJ. Treatment of obese children with and without their mothers: changes in weight and blood pressure. Pediatrics. 1983;71(4):515–523. 6835735

[8] Israel AC, Silverman WK, Solotar LC. An investigation of family influences on initial weight status, attrition, and treatment outcome in a childhood obesity program. Behav Ther. 1986;17:131–143.

[9] Loveman E, Al-Khudairy L, Johnson RE, Parent-only interventions for childhood overweight or obesity in children aged 5 to 11 years. Cochrane Database Syst Rev. 2015;2015(12):CD012008. 26690844 10.1002/14651858.CD012008 PMC8761478

[10] Colquitt JL, Loveman E, O’Malley C, Diet, physical activity, and behavioural interventions for the treatment of overweight or obesity in preschool children up to the age of 6 years. Cochrane Database Syst Rev. 2016;3(3):CD012105. 26961576 10.1002/14651858.CD012105 PMC6669248

[11] Epstein LH, Paluch RA, Roemmich JN, Beecher MD. Family-based obesity treatment, then and now: twenty-five years of pediatric obesity treatment. Health Psychol. 2007;26(4):381–391. 17605557 10.1037/0278-6133.26.4.381 PMC2387251

[12] Pratt KJ, Lazorick S, Eneli I, Collier DN, Skelton JA. Providers’ involvement of blended families in pediatric weight management programs. Fam Syst Health. 2019;37(4):320–327. 31613126 10.1037/fsh0000446

[13] Cain KS, Cohen GM, Skelton JA, Crawford LV, Brown CL. Assessing parents’ communication of weight and weight management from clinic to home. Child Obes. 2020;16(7):510–519. 32744874 10.1089/chi.2019.0207 PMC7575346

[14] Graham HB. Corpulence in childhood and adolescence; a clinical study. Med J Aust. 1947;2(22):649–658. [Discussion 672]. 18919192

[15] Illingworth RS. Childhood obesity. Practitioner. 1970;205(226): 201–202. 5480511

[16] Rayner PH, Court JM. Proceedings: effect of dietary restriction and anorectic drugs on linear growth in childhood obesity. Arch Dis Child. 1974;49(10):822–823. 10.1136/adc.49.10.822-c PMC1649195 4429382

[17] Charney E, Goodman HC, McBride M, Lyon B, Pratt R. Childhood antecedents of adult obesity. Do chubby infants become obese adults? N Engl J Med. 1976;295(1):6–9. 1272299 10.1056/NEJM197607012950102

[18] Jordan HA, Levitz LS. Behavior modification in the treatment of childhood obesity. Curr Concepts Nutr. 1975;3:141–150. 1093808

[19] Wing RR, Marcus MD, Epstein LH, Jawad A. A “family-based” approach to the treatment of obese type II diabetic patients. J Consult Clin Psychol. 1991;59(1):156–162. 2002132 10.1037//0022-006x.59.1.156

[20] Epstein LH, Wing RR, Steranchak L, Dickson B, Michelson J. Comparison of family-based behavior modification and nutrition education for childhood obesity. J Pediatr Psychol. 1980;5(1):25–36. 7452420 10.1093/jpepsy/5.1.25

[21] Epstein LH, Wing RR, Koeske R, Valoski A. Long-term effects of family-based treatment of childhood obesity. J Consult Clin Psychol. 1987;55(1):91–95. 3106445 10.1037//0022-006x.55.1.91

[22] Brownell KD. New developments in the treatment of obese children and adolescents. Res Publ Assoc Res Nerv Ment Dis. 1984;62:175–183. 6695113

[23] Brownell KD, Wadden TA. Confronting obesity in children: behavioral and psychological factors. Pediatr Ann. 1984;13(6):473–478, 480. 6462794

[24] Israel AC, Stolmaker L, Andrian CA. The effects of training parents in general child management skills on a behavioral weight loss program for children. Behav Ther. 1985;16(2):169–180.

[25] Graves T, Meyers AW, Clark L. An evaluation of parental problem-solving training in the behavioral treatment of childhood obesity. J Consult Clin Psychol. 1988;56(2):246–250. 3372833 10.1037//0022-006x.56.2.246

[26] Epstein LH, Valoski A, Wing RR, McCurley J. Ten-year follow-up of behavioral, family-based treatment for obese children. Jama. 1990; 264(19):2519–2523. 2232019

[27] Epstein LH, Valoski A, Wing RR, McCurley J. Ten-year outcomes of behavioral family-based treatment for childhood obesity. Health Psychol. 1994;13(5):373–383. 7805631 10.1037//0278-6133.13.5.373

[28] Flodmark CE, Ohlsson T, Ryden O, Sveger T. Prevention of progression to severe obesity in a group of obese schoolchildren treated with family therapy. Pediatrics. 1993;91(5):880–884. 8474806

[29] Golan M, Weizman A, Apter A, Fainaru M. Parents as the exclusive agents of change in the treatment of childhood obesity. Am J Clin Nutr. 1998;67(6):1130–1135. 9625084 10.1093/ajcn/67.6.1130

[30] Boutelle KN, Cafri G, Crow SJ. Parent-only treatment for childhood obesity: a randomized controlled trial. Obesity (Silver Spring). 2011; 19(3):574–580. 20966907 10.1038/oby.2010.238 PMC4008332

[31] Boutelle KN, Cafri G, Crow SJ. Parent predictors of child weight change in family based behavioral obesity treatment. Obesity (Silver Spring). 2012;20(7):1539–1543. 22421896 10.1038/oby.2012.48 PMC4456177

[32] Chai LK, Collins C, May C, Brain K, Wong See D, Burrows T. Effectiveness of family-based weight management interventions for children with overweight and obesity: an umbrella review. JBI Database System Rev Implement Rep. 2019;17(7):1341–1427. 10.11124/JBISRIR-2017-003695 31021970

[33] Barlow SE, Dietz WH. Obesity evaluation and treatment: expert committee recommendations. The Maternal and Child Health Bureau, Health Resources and Services Administration and the Department of Health and Human Services. Pediatrics. 1998;102(3):E29. 9724677 10.1542/peds.102.3.e29

[34] Barlow SE. Expert committee recommendations regarding the prevention, assessment, and treatment of child and adolescent overweight and obesity: summary report. Pediatrics. 2007;120(suppl 4): S164–S192. 18055651 10.1542/peds.2007-2329C

[35] Kang Sim DE, Strong DR, Manzano MA, Rhee KE, Boutelle KN. Evaluation of dyadic changes of parent–child weight loss patterns during a family-based behavioral treatment for obesity. Pediatr Obes. 2020; 15(6):e12622. 32048808 10.1111/ijpo.12622 PMC9261271

[36] Dickens CE, Safer DL, Runfola CD, Gibbs EL, Welch H, Sadeh-Sharvit S. The offspring of parents undergoing a weight loss surgery: a systematic review. Surg Obes Relat Dis. 2020;16(6):806–815. 32334972 10.1016/j.soard.2020.02.006

[37] Song M, Lee CS, Lyons KS, Stoyles S, Winters-Stone KM. Assessing the feasibility of parent participation in a commercial weight loss program to improve child body mass index and weight-related health behaviors. SAGE Open Med. 2018;6:2050312118801220. 10.1177/2050312118801220 PMC6170954 30302248

[38] Pham SB, Skelton JA, Pratt K, Lewis KH, Brown CL. Examining the effect of parent participation in an adult weight management program on changes in children’s weight. Clin Obes. 2023;13(2):e12583. 36759742 10.1111/cob.12583 PMC9992107

[39] Berge JM, Meyer C, MacLehose RF, Crichlow R, Neumark-Sztainer D. All in the family: correlations between parents’ and adolescent siblings’ weight and weight-related behaviors. Obesity (Silver Spring). 2015;23(4):833–839. 25820257 10.1002/oby.21036 PMC4380227

[40] Berge JM, Meyer C, MacLehose RF, Loth K, Neumark-Sztainer D. Do parents treat siblings similarly or differently with regard to feeding practices, weight-related conversations, and support for physical activity? An exploratory analysis. Child Obes. 2016;12(2):87–93. 26699095 10.1089/chi.2015.0049 PMC4817570

[41] Berge JM, Tate AD, Trofholz A, Conger K, Neumark-Sztainer D. Sibling eating behaviours and parental feeding practices with siblings: similar or different? Public Health Nutr. 2016;19(13):2415–2423. 27122059 10.1017/S1368980016000860 PMC5940331

[42] Berge JM, Hanson-Bradley C, Tate A, Neumark-Sztainer D. Do parents or siblings engage in more negative weight-based talk with children and what does it sound like? A mixed-methods study. Body Image. 2016;18:27–33. 27236475 10.1016/j.bodyim.2016.04.008 PMC5012935

[43] Kitzmann KM, Beech BM. Family-based interventions for pediatric obesity: methodological and conceptual challenges from family psychology. J Fam Psychol. 2006;20(2):175–189. 16756393 10.1037/0893-3200.20.2.175

[44] Nowicka P, Flodmark CE. Family therapy as a model for treating childhood obesity: useful tools for clinicians. Clin Child Psychol Psychiatry. 2011;16(1):129–145. 20650975 10.1177/1359104509355020

[45] Nowicka P, Pietrobelli A, Flodmark CE. Low-intensity family therapy intervention is useful in a clinical setting to treat obese and extremely obese children. Int J Pediatr Obes. 2007;2(4):211–217. 17852553 10.1080/17477160701379810

[46] Skelton JA, Buehler C, Irby MB, Grzywacz JG. Where are family theories in family-based obesity treatment? Conceptualizing the study of families in pediatric weight management. Int J Obes (Lond). 2012; 36(7):891–900. 22531090 10.1038/ijo.2012.56 PMC3977510

[47] Pratt KJ, Skelton JA. Family functioning and childhood obesity treatment: a family systems theory-informed approach. Acad Pediatr. 2018;18:620–627. 29654905 10.1016/j.acap.2018.04.001 PMC8111666

[48] Smith JD, Montano Z, Maynard A, Miloh T. Family functioning predicts body mass index and biochemical levels of youths with nonalcoholic fatty liver disease. J Dev Behav Pediatr. 2017;38(2):155–160. 27984419 10.1097/DBP.0000000000000379

[49] Halliday JA, Palma CL, Mellor D, Green J, Renzaho AM. The relationship between family functioning and child and adolescent overweight and obesity: a systematic review. Int J Obes (Lond). 2014;38(4): 480–493. 24232501 10.1038/ijo.2013.213

[50] Zeller MH, Hunsaker S, Mikhail C, Family factors that characterize adolescents with severe obesity and their role in weight loss surgery outcomes. Obesity (Silver Spring). 2016;24(12):2562–2569. 27753228 10.1002/oby.21676 PMC5379472

[51] Skelton JA, Van Fossen C, Harry O, Pratt KJ. Family dynamics and pediatric weight management: putting the family into family-based treatment. Curr Obes Rep. 2020;9(4):424–441. 33108634 10.1007/s13679-020-00407-9

[52] Nowicka P, Flodmark CE. Family in pediatric obesity management: a literature review. Int J Pediatr Obes. 2008;3(suppl 1):44–50. 18278632 10.1080/17477160801896994

[53] Bowen M. The use of family theory in clinical practice. Compr Psychiatry. 1966;7:345–374. 5922263 10.1016/s0010-440x(66)80065-2

[54] White JM, Klein DM. The systems framework. Family Theories. 3rd ed. Sage Publications; 2008:151–177.

[55] Epstein NB, Baldwin LM, Bishop DS. The McMaster family assessment device. J Marital Fam Ther. 1983;9(2):171–180.

[56] Ryan CE, Epstein NB, Keitner GI, Miller IW, Bishop DS. Evaluating and Treating Families: The McMaster Approach. Routledge; 2005.

[57] Sung-Chan P, Sung YW, Zhao X, Brownson RC. Family-based models for childhood-obesity intervention: a systematic review of randomized controlled trials. Obes Rev. 2013;14(4):265–278. 23136914 10.1111/obr.12000

[58] Slesnick N, Zhang J. Family systems therapy for substance-using mothers and their 8- to 16-year-old children. Psychol Addict Behav. 2016;30(6):619–629. 27454370 10.1037/adb0000199 PMC5025363

[59] Riley AR, Duke DC, Freeman KA, Hood KK, Harris MA. Depressive symptoms in a trial behavioral family systems therapy for diabetes: a post hoc analysis of change. Diabetes Care. 2015;38(8):1435–1440. 26015558 10.2337/dc14-2519

[60] Dallacker M, Hertwig R, Mata J. The frequency of family meals and nutritional health in children: a meta-analysis. Obes Rev. 2018;19(5): 638–653. 29334693 10.1111/obr.12659

[61] do Amaral EMGR, Silva PO, Nakabayashi J, Bandeira MV, Toral N, Monteiro R. Family meal frequency and its association with food consumption and nutritional status in adolescents: a systematic review. PLoS One. 2020;15(9):e0239274. 32946506 10.1371/journal.pone.0239274 PMC7500660

[62] Berge JM, Wall M, Hsueh TF, Fulkerson JA, Larson N, Neumark-Sztainer D. The protective role of family meals for youth obesity: 10-year longitudinal associations. J Pediatr. 2015;166(2):296–301. 25266343 10.1016/j.jpeds.2014.08.030 PMC4308550

[63] Glanz K, Metcalfe JJ, Folta SC, Brown A, Fiese B. Diet and health benefits associated with in-home eating and sharing meals at home: a systematic review. Int J Environ Res Public Health. 2021;18(4):1577. 33562357 10.3390/ijerph18041577 PMC7915304

[64] Goldfarb S, Tarver WL, Sen B. Family structure and risk behaviors: the role of the family meal in assessing likelihood of adolescent risk behaviors. Psychol Res Behav Manag. 2014;7:53–66. 24627645 10.2147/PRBM.S40461 PMC3931580

[65] Skeer MR, Ballard EL. Are family meals as good for youth as we think they are? A review of the literature on family meals as they pertain to adolescent risk prevention. J Youth Adolesc. 2013;42(7):943–963. 23712661 10.1007/s10964-013-9963-z

[66] Larson RW, Branscomb KR, Wiley AR. Forms and functions of family mealtimes: multidisciplinary perspectives. New Dir Child Adolesc Dev. 2006; Spring(111):1–15. 10.1002/cd.152 16646496

[67] Knowler WC, Barrett-Connor E, Fowler SE, Reduction in the incidence of type 2 diabetes with lifestyle intervention or metformin. N Engl J Med. 2002;346(6):393–403. 11832527 10.1056/NEJMoa012512 PMC1370926

[68] Vitolins MZ, Isom SP, Blackwell CS, The healthy living partnerships to prevent diabetes and the diabetes prevention program: a comparison of year 1 and 2 intervention results. Transl Behav Med. 2017;7(2):371–378. 27796775 10.1007/s13142-016-0447-z PMC5526803

[69] Ash T, Agaronov A, Young T, Aftosmes-Tobio A, Davison KK. Family-based childhood obesity prevention interventions: a systematic review and quantitative content analysis. Int J Behav Nutr Phys Act. 2017;14(1):113. 28836983 10.1186/s12966-017-0571-2 PMC5571569

[70] Davison GM, Fowler LA, Ramel M, Racial and socioeconomic disparities in the efficacy of a family-based treatment programme for paediatric obesity. Pediatr Obes. 2021;16(10):e12792. 33847074 10.1111/ijpo.12792 PMC8440359

